# INNOVATIVE APPROACHES TO ADDRESSING LONELINESS AND PROMOTING AGING IN COMMUNITY

**DOI:** 10.1093/geroni/igad104.0739

**Published:** 2023-12-21

**Authors:** Su-I Hou, Li-Fan Liu, Carol Ma

## Abstract

Research has shown that older adults who are lonely and socially isolated are at increased risk for various negative health outcomes. This symposium brings together leading experts across the globe in the fields of gerontology, public health, and social work to share innovative approaches to addressing loneliness and fostering connections. The symposium will begin with Dr. Ma from the National Singapore University of Social Sciences (SUSS) to share the voice of seniors on digital inclusion for a healthy community in Singapore. Dr. Shapira from Ben-Gurion University of the Negev, Israel, and her research team will discuss a 7-session digital intervention to promote effective coping and alleviate depression and loneliness among older adults in Israel. The symposium will then examine community-based efforts, with Dr. Hou from The University of Central Florida (UCF) in the United States discussing the role of healthcare providers in screening and identifying loneliness and examining communication and comfortability in conducting loneliness screening among older patients. Lastly, Dr. Kuo from Chung-Shan Medical University (CSMU) will introduce the five domains of living a good life and how older adults responded to these topics to prepare themselves for better aging in the community. The presenters will discuss strategies for fostering community-based support networks to address community-dwelling older adults’ physical, social, and emotional needs. This global symposium will also examine the role of technology in supporting community-based efforts, including using virtual communication platforms, online resources, and digital tools to promote community engagement and connection. This is an International Comparisons of Healthy Aging Interest Group Sponsored Symposium. This is a collaborative symposium between the Aging Among Asians and International Comparisons of Healthy Aging Interest Groups.

